# From Biobank to Bedside: A Pilot Study on Returning Medically Actionable BRCA1/2 Results in Qatar’s Precision Medicine Landscape

**DOI:** 10.3390/biomedicines13123047

**Published:** 2025-12-11

**Authors:** Salha Bujassoum Al Bader, Hind Habish, Hajer Almulla, Fatemeh Abbaszadeh, Mariem Sidenna, Tasnim Fadl, Mohamed Alvi, Marwa Eldeeb, Huda Farah, Amal Elfatih, Radja Messai Badji, Lotfi Chouchane, Nahla Afifi, Said Ismail, Reem Alsulaiman, Wadha Al-Muftah

**Affiliations:** 1 Hamad Medical Corporation, Doha P.O. Box 3050, Qatar; 2 Qatar Precision Health Institute, Doha P.O. Box 5825, Qataraeabdelmageed@qf.org.qa (A.E.); rbadji@qf.org.qa (R.M.B.);; 3 Weill Cornell Medicine–Qatar, Doha P.O. Box 24144, Qatar; 4 College of Health and Life Sciences, Hamad Bin Khalifa University, Doha P.O. Box 34110, Qatar

**Keywords:** *BRCA*, cancer surveillance, actionable findings, biobank, genome, Qatar

## Abstract

**Background:** Hereditary breast and ovarian cancer is an inherited condition caused by pathogenic (P) or likely pathogenic (LP) variants in the *BRCA1* and *BRCA2* genes. Population-level sequencing allows for the identification of asymptomatic genotype-positive participants (GPPs) before disease onset. This study assessed the feasibility and impact of returning clinically relevant *BRCA* results to participants at the Qatar Precision Health Institute (QPHI). **Methods:** We established a structured framework to identify and refer asymptomatic individuals who were found to carry P/LP variants in *BRCA* among 6142 QPHI participants. The process integrated genomic analysis, participant recontact, counseling, referral, variants validation, and personalized risk-reducing strategies. **Results:** Six variants (four *BRCA1*, two *BRCA2*) were validated in ten GPPs with a median age of 48 years (IQR: 40.5–56). Eight variants were confirmed through Sanger sequencing in a CAP-accredited laboratory at Hamad Medical Corporation. All eligible participants were referred for counseling and personalized clinical management. Four men initiated breast and prostate cancer surveillance, while four women pursued breast and ovarian surveillance. One asymptomatic GPP underwent prophylactic salpingo-oophorectomy, revealing early-stage ovarian cancer. Cascade testing identified 20 additional GPPs and, in one asymptomatic relative, facilitated the detection of early-stage uterine cancer. The genetic testing acceptability rate was 0.77 (95% CI: 0.46–0.94), with a 100% adherence to surveillance at 12- and 24-month follow-ups. **Conclusions:** This pilot demonstrates the feasibility and clinical utility of returning actionable *BRCA1/2* findings and represents the first initiative in an Arabic population to implement the return of medically actionable *BRCA* results from a population-based biobank.

## 1. Background

Genome sequencing increases the likelihood of detecting secondary findings (SFs), which are results not directly related to the primary indications for genetic testing but that have an established clinical actionability. The approach to reporting these findings has transitioned from initial recommendations to the establishment of well-defined and structured guidelines [1,2,3,4,5,6,7]. The American College of Medical Genetics and Genomics (ACMG) recommends the analysis and reporting of pathogenic (P) and likely pathogenic (LP) variants in 81 genes, including *BRCA1* and *BRCA2*, as these genes are deemed to be medically actionable and have available therapeutic and preventive interventions [8]. Given the complexity and potential health impact of returning actionable SFs, the broader utilization of genetic testing into routine clinical care raises several technical, logistical, and ethical challenges [9]. Addressing these challenges requires the integration of multidisciplinary efforts to effectively manage the clinical implications of these findings.

Individuals carrying germline P/LP variants in the *BRCA1* and *BRCA2* genes have a cumulative lifetime risk of female breast cancer that is up to 80% and ovarian cancer risk up to 60% by age 80. Moreover, pathogenic variants in these genes can increase the male risk of breast and prostate cancer [10]. However, previous data indicate that *BRCA1/2* testing provides significant benefits, particularly when coupled with risk-reducing strategies such as increased surveillance or prophylactic surgeries. For example, a prophylactic mastectomy can reduce breast cancer risk by more than 90% [11]. In Qatar, breast cancer remains the most diagnosed cancer among women, accounting for 39% of all cancer cases recorded in 2022, and has the highest mortality rate (11.4%) compared to other cancer types [12]. To address this, Qatar has implemented a national breast cancer screening program, offering population-wide screenings every three years for average-risk women aged 45 to 69. This initiative has proven effective in detecting early-stage breast cancer within a standard-risk population.

To improve early cancer detection among high-risk individuals, Hamad Medical Corporation (HMC) established a cancer genetic program in 2013 at the National Center for Cancer Care and Research (NCCCR). This program offers genetic counseling and high-risk surveillance to identify patients with an elevated lifetime risk of developing various cancers, as well as individuals already affected. The program offers genetic assessment, genetic testing, and risk-reducing strategies for high-risk individuals, as well as therapeutic measures and reproductive options based on the identified syndromes. Additionally, the program also offers cascade testing for family members, as well as personalized risk-reducing strategies [13]. Recently, HMC established the Centre of Clinical Precision Medicine and Genomics (CCPMG) in 2023 to serve as a model for multidisciplinary collaboration and to integrate genomic science into clinical workflows for delivering innovative and patient-centered care. The center features a one-stop clinic that combines clinical assessment, molecular laboratory testing, clinical management, and genomics-based lifestyle interventions under one roof, ensuring precise diagnoses, tailored treatments, and improved patient outcomes.

The Qatar Precision Health Institute (QPHI) is a national research center that integrates genome sequencing data from the Qatar Genome Program with comprehensive phenotypic data and biological samples from the Qatar Biobank to advance precision health [14]. In collaboration with the NCCCR program at HMC, we developed a communication and referral plan to evaluate the feasibility and clinical impact of delivering medically actionable *BRCA1* and *BRCA2* results to QPHI participants. Utilizing whole-genome sequencing (WGS) data from 6142 participants, we established a framework for returning medically actionable *BRCA1/2* findings and assessed the feasibility of integrating genetic testing with clinical surveillance to translate risk-reducing interventions from research to clinical practice.

## 2. Methods

### 2.1. The Qatar Precision Health Institute Cohort

The Qatar Biobank is a prospective, longitudinal, population-based study designed to generate extensive data from clinical assessments of Qataris and long-term residents of Qatar aged 18 years and older. Participants are followed up with every five years, and the data are integrated with genome sequencing data from the Qatar Genome Program [14,15]. All participants signed a general consent form allowing their data to be used anonymously for research purposes and permitting recontact for follow-up visits. The pilot study was approved by the Qatar Biobank Institutional Review Board (QF-QBB-RES-ACC-0241) and by the ethical committee of Hamad Medical Corporation (HMC) (MRC-01-20-1148).

This study utilized genome sequencing data from 6142 participants recruited between December 2012 and August 2017. Detailed information about the QPHI cohort, phenotypic data, and genome sequencing is available in previous publications [14,15,16]. In brief, QPHI recruited participants who then attended an assessment session involving physical measurements and clinical tests. Standardized questionnaires were used to collect phenotypic data, capturing information on lifestyle and medical history. Biological samples (blood, saliva, and urine) were provided and stored at –80 °C in liquid nitrogen. DNA was extracted from peripheral blood and sequenced on the HiSeq X Ten (Illumina, San Diego, CA, USA), with a minimum coverage of 30×. After quality control, the reads were aligned to the GRCh37 reference genome using bwa.kit (version 0.7.12). Variant calling was performed using the Genome Analysis Toolkit 3.4 best practices.

### 2.2. Definition of BRCA1/2 Actionable Findings

A medically actionable finding was defined as a P or LP variant in the *BRCA1* or *BRCA2* genes. These variants had associated phenotypes unrelated to the primary test indication and were recommended for reporting to participants according to ACMG guidelines [8]. In addition to the biobank data, our analysis incorporated the bioinformatic annotation of *BRCA1* and *BRCA2* gene results from a publication by the Qatar Genome Program research consortium, authored by Saad and colleagues [16]. The final annotated variant list included any variant classified as P or LP in either ClinVar or CharGer [17], without any conflicting interpretations at a maximum allele frequency of less than 1%.

As the list of P/LP variants in *BRCA1/2* genes provided by Saad and the team had been classified in research settings rather than clinics, each variant underwent a secondary review to generate a final list of P/LP variants that followed the ACMG/AMP classification criteria [18]. Participants with variants from this list were identified as potentially eligible for the pilot study.

### 2.3. Eligibility Criteria and Return of Results Workflow

In June 2019, the QPHI study protocol was updated to include the return of results, and only participants who opted for recontact were selected. Initially, de-identified codes were used to anonymize all participants in the research study conducted by Saad and the team, ensuring confidentiality. These participants were later re-identified at QPHI to access their electronic medical records (EMRs) and exclude those with a known medical diagnosis of breast and/or ovarian cancer, participants enrolled in the high-risk surveillance program, or participants who had previously received positive genetic test results for *BRCA1/2* from other sources.

The potentially eligible participants were subsequently contacted during their follow-up visits to introduce the pilot study. A detailed family history was obtained from all eligible participants and was designated as strongly positive if a direct family member had been diagnosed with breast or ovarian cancer, or if they exhibited symptoms consistent with these cancers. A fresh blood sample (10 mL) was collected and sent to the clinically certified, CAP-accredited Diagnostics Genomic Division laboratory at HMC to confirm the presence of P/LP variants in *BRCA1/2* genes through Sanger sequencing.

### 2.4. Genetic Counseling and Downstream Surveillance

Two dedicated genetic counselors were assigned to conduct counseling sessions for all eligible participants. In the first session, which occurred before the confirmatory Sanger sequencing test, the implications of *BRCA1/2* actionable findings were explained, and any refusals to participate in the pilot study were documented. The second session took place after the presence of P/LP variants in *BRCA1/2* was confirmed at the HMC CAP-accredited laboratory. During this session, the results were communicated by a clinical cancer genetic counselor to both the participants and their eligible family members, along with the potential health implications.

Based on the results of the confirmatory genetic test for *BRCA1/2* variants, participants were classified into three groups. The first group consisted of participants who tested positive for P/LP variants in *BRCA1/2*, irrespective of their family history of associated cancers. The second group included those who tested negative but had a strong family history of breast or ovarian cancer. Participants in these two groups were referred to the cancer genetics and high-risk surveillance program at HMC for further assessment. The third group included participants who tested negative in the confirmatory genetic test and had no family history of breast or ovarian cancer. Participants from the third group were referred to a genetic counselor for reassurance, with no further action required.

The surveillance strategies at the cancer genetics program were guided by local protocols and the National Comprehensive Cancer Network clinical practice guidelines [19]. The program offered two breast risk-reduction strategies. The first strategy involved high-risk surveillance for women, including clinical breast examinations and alternating MRI and mammogram radiological imaging every six months. The second option included risk-reducing bilateral mastectomies with immediate breast oncoplastic surgeries. For men, high-risk surveillance included clinical breast examinations, regular prostate-specific antigen (PSA) testing at defined intervals, digital rectal examinations, and imaging as needed.

For ovarian cancer, participants were offered either surveillance with pelvic or transvaginal ultrasounds and CA125 tumor marker testing or risk-reducing bilateral salpingo-oophorectomies, based on patient preference and after the completion of family planning.

### 2.5. Assessment of Acceptability and Adherence Rates

To evaluate the acceptability and adherence to the cancer surveillance and clinical intervention for *BRCA1/2* carriers, clinical and radiological data were collected from participants’ medical records at three time points: at the uptake of surveillance (defined as the first surveillance following the genetic confirmatory test), and then at 12 and 24 months after enrollment in the pilot study. In this study, surveillance data referred to HMC-EMRs for breast, ovarian, or prostate cancer, including clinical, radiological, and tumor marker tests, which were recorded in real time (or near real time).

Acceptability for surveillance was defined as the proportion of eligible participants who were invited to the study and underwent clinical assessment or testing at the uptake of surveillance. Adherence was assessed by measuring the prevalence of participants who remained current with radiological testing at both 12 and 24 months after enrolling in the pilot study.

### 2.6. Statistical Analysis

Statistical analyses were conducted using R software (version 4.4.2). Descriptive statistics were used to summarize the data. Continuous variables were reported using the median with interquartile range (IQR). To analyze acceptability and adherence, proportions and their 95% confidence intervals (CIs) were calculated using a one-proportion Z-test.

## 3. Results

### 3.1. Recontact and Return of BRCA1/2 Results

**Figure 1:**
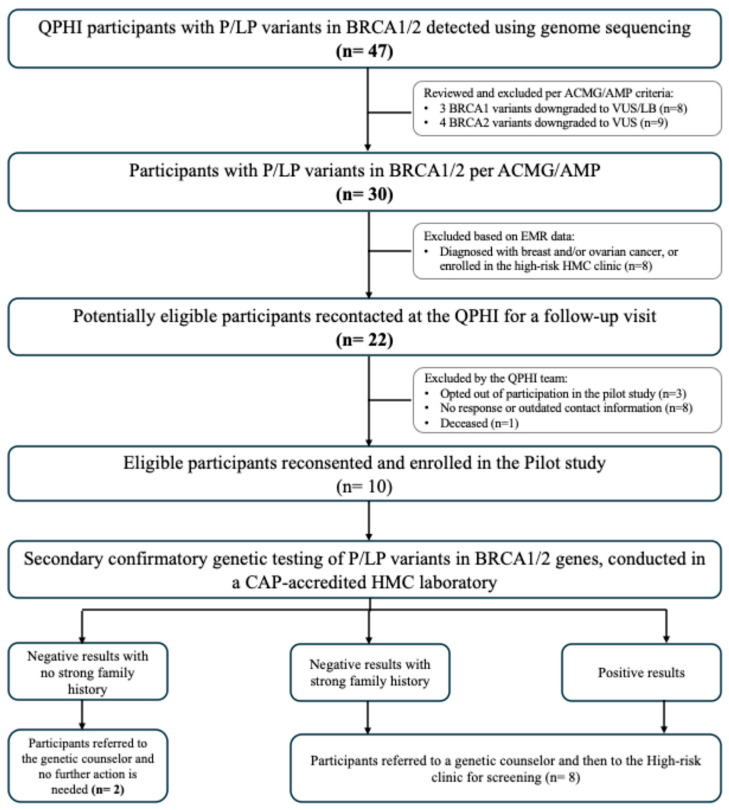
Results from piloting the return of BRCA1/2 actionable findings workflow.

A description of the workflow design and the number of participants at each stage is illustrated in Figure 1. Initially, we identified 47 genotype-positive participants (GPPs) carrying 22 P/LP variants in the *BRCA1/2* genes. These included 10 variants found in *BRCA1* (carried by 24 GPPs) and 12 variants in *BRCA2* (carried by 23 GPPs) (Supplementary Table S1). All 47 participants opted for recontact as part of their informed consent process. Saad and colleagues initially classified three *BRCA1* variants (in eight GPPs) and four *BRCA2* variants (in nine GPPs) as P/LP. However, a subsequent review, based on ACMG/AMP guidelines, downgraded these variants to variants of uncertain significance (VUS) or likely benign. As a result, 17 participants carrying these variants were excluded from the list of potentially eligible participants.

The review of EMR data led to the exclusion of eight more participants due to a prior diagnosis of breast or ovarian cancer, or enrollment in the high-risk medical oncology clinic. Of the 22 potentially eligible participants, 12 were further excluded for the following reasons: opting out of the pilot study (*n* = 3), failure to respond or outdated contact information (*n* = 8), and confirmation of death at the time of recontact (*n* = 1). The remaining 10 eligible participants reconsented and enrolled in the pilot study.

### 3.2. Validated BRCA1/2 Actionable Findings

We included ten participants in the pilot study, carrying six variants. Four variants were identified in *BRCA1* and two in the *BRCA2* gene (Table 1). In *BRCA1*, two participants were found to carry the c.4787C>A (p.Ser1596Ter) variant, which was validated through confirmatory Sanger sequencing at the HMC laboratory. Additionally, two participants (siblings, #4 and #10) were confirmed to carry the c.1365dup (p.Ile456fs), classified as likely pathogenic, while another participant carried the c.4065_4068del (p.Asn1355fs) variant, classified as pathogenic, in the *BRCA1* gene. Additionally, Sanger sequencing confirmed the pathogenic variant c.4211_4215del (p.Ser1404Ter) in two *BRCA2* participants, and the likely pathogenic variant c.-39-1G>C in one participant.

The Sanger sequencing test confirmed the presence of the c.4096+1G>C variant in *BRCA1* carried by two GPPs (participants #6 and #7). However, the clinical scientist team reclassified this variant as VUS because computational analysis predicted that c.4096+1G>C would likely disrupt the natural splice donor site of intron 11 in *BRCA1*, but this prediction was not supported by published functional studies. Therefore, its clinical significance remained uncertain at the time of analysis.

### 3.3. Clinical Implications of BRCA1/2 Genetic Findings

The ten pilot study participants (six females and four males) had a median age of 48 years (IQR: 40.5–56) (Table 2). Only four participants (including two siblings, #4 and #10) reported a strong family history of breast or ovarian cancer at the time of enrollment. Detailed information on family history and participant pedigrees is provided in Supplementary Figure S1. All ten participants underwent clinical-grade Sanger sequencing, and their results were returned by the genetic counselor. None of the participants requested a referral to the HMC psychiatric department for anxiety management.

Among the ten eligible participants in the pilot study, two female participants (#6 and #7) had their results validated through Sanger sequencing and reclassified as VUS. Consequently, the genetic counselor addressed their general risk for breast and ovarian cancer and educated them on breast cancer screening through the national program. The remaining eight participants had their P/LP variants validated at the HMC laboratory (five GPPs in *BRCA1* and three GPPs in *BRCA2*) and were referred to the cancer genetics and high-risk surveillance program. None of the pilot study participants had a negative confirmatory genetic test alongside a strong family history of breast or ovarian cancer.

At the cancer genetics and high-risk surveillance program, risk-reducing strategies were offered. Among the eight participants with confirmed *BRCA1/2* P/LP variants, four males (participants #1, 2, 3, and #8) were offered surveillance that included breast examination and imaging, as well as follow-ups for prostate cancer at the urology–oncology department, which involved blood tests and clinical assessments. Three female participants (# 4, #9, and #10) were offered breast cancer surveillance, which included alternating breast MRI and mammography or ultrasound, in addition to ovarian cancer surveillance, which involved pelvic ultrasound and CA-125 blood tests. Participant #5 underwent breast cancer surveillance; however, she opted for prophylactic BSO, which revealed an early-stage ovarian cancer (high-grade invasive serous carcinoma of the fallopian tube with serous tubal intraepithelial carcinoma, FIGO stage IA). This was followed by adjuvant chemotherapy with excellent clinical outcomes, and the patient has remained disease-free post-surgery, with no recurrence reported at her last follow-up.

Genetic counseling was offered to all first-degree relatives of the index cases at HMC. Twenty family members agreed to undergo cascade genetic testing via Sanger sequencing and were found to carry P/LP variants in the *BRCA1/2* genes. They were subsequently offered personalized risk-reducing strategies. All 13 female family members underwent breast cancer surveillance, which included imaging every six months (mammography and MRI), in addition to ovarian cancer surveillance through pelvic or transvaginal ultrasounds with CA125 testing, based on factors such as age, patient preference, and family planning status. One family member (sister of index case #5) chose to undergo a total abdominal hysterectomy with bilateral salpingo-oophorectomy and was found to have early-stage uterine cancer (serous endometrioid carcinoma). All seven male carriers underwent breast cancer surveillance through clinical examination and prostate cancer surveillance, which included PSA testing and clinical examinations, with imaging performed as indicated.

### 3.4. Participants’ Acceptability and Adherence Rates

**Figure 2:**
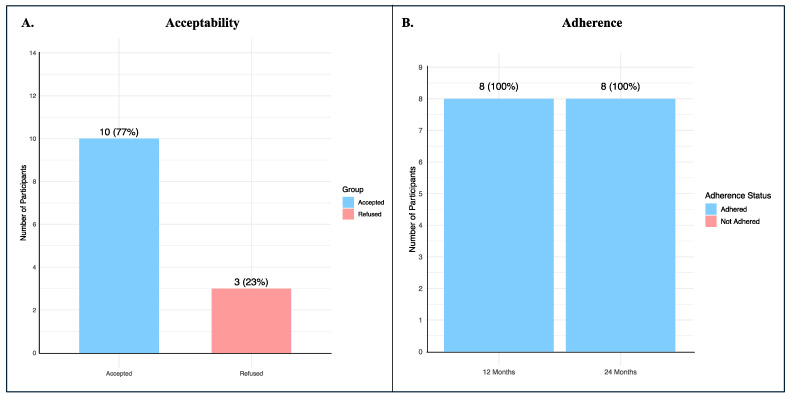
Acceptability and adherence of BRCA1/2 participants. (**A**) The proportion of individuals who agreed to participate in the pilot study following introduction at QPHI, with the calculated acceptability rate; (**B**) The proportion of enrolled participants who remained on surveillance at 12 and 24 months after uptake, demonstrating full adherence at both time points.

A total of 13 potential participants were seen at QPHI, where the pilot study was introduced, and consent for variant validation through Sanger sequencing was requested. Only three participants refused to participate in the pilot study. The acceptability rate at the uptake surveillance was 0.77 (95% CIs: 0.54–0.998) (Figure 2A).

We calculated the proportion of participants who were currently on surveillance 12 and 24 months after the uptake surveillance, and the adherence rate was 100% at both time points (Figure 2B).

## 4. Discussion

Genome sequencing can offer a wide range of health benefits to participants in research studies by returning medically actionable results and offering the early identification of at-risk individuals, therefore allowing for personalized risk-reducing strategies and reducing morbidity and mortality for high-risk groups [20,21]. Limited resources and capacity represent challenges for effective clinical management, highlighting the need for structured workflows to maximize the health benefits of identifying medically actionable findings [16,22,23]. A growing number of national genomics programs, especially from underrepresented populations, are providing opportunities to guide the system-wide changes required for the clinical implementation of genomics research [24].

A previous study in Qatar evaluated the impact of *BRCA1* and *BRCA2* pathogenic mutations on breast cancer aggressiveness in carriers versus non-carriers, involving 82 women diagnosed with breast cancer at the age of 50 or younger [25]. The results suggested that *BRCA1/2* mutations were associated with breast cancer that exhibited more aggressive behavior, particularly in younger age groups. Additionally, patients with these mutations tended to present with more advanced stages of disease compared to those without pathogenic mutations in the *BRCA1/2* genes, who exhibited a less aggressive cancer. These findings emphasized the importance of establishing a tailored workflow for participants in the population-based Qatar biobank to bridge the gap between research and clinical care for participants carrying P/LP variants in *BRCA1/2* genes and to ensure appropriate support and disclosure of these findings to participants.

In this pilot study, we established a return of results workflow for *BRCA1/2* medically actionable genetic findings emerging from the research-grade whole-genome sequencing of 6142 participants. This workflow was developed sequentially to assess the feasibility of integrating genetic testing into routine clinical high-risk surveillance for breast, ovarian, and prostate cancer, and to address potential challenges. Using this designed workflow, which integrates multidisciplinary approaches, including genomic data analysis, variant classification and validation, participant recontact, and proper counseling, we were able to return medically actionable *BRCA1/2* findings to ten participants and to observe how these findings can influence their management plans and future risk.

A key contributor to the success of the pilot was the inclusion of structured pre- and post-test genetic counseling. The pre-test sessions ensured that participants received clear and accessible explanations about the purpose, scope, benefits, and limitations of the genetic testing, enabling informed decision-making and reducing uncertainty. Post-test counseling provided individualized interpretation of results, answered participants’ questions, and guided appropriate clinical follow-ups. Importantly, the post-test counseling also included counseling on cascade genetic testing for at-risk relatives. This led to the testing and identification of 20 family members carrying *BRCA1/2* pathogenic or likely pathogenic variants, demonstrating the program’s meaningful clinical impact at both the patient and family levels.

The acceptability rate for returning *BRCA1/2* genetic information and referring to a high-risk genetic clinic among pilot study participants was 77% (95% CIs: 0.46–0.94). Although all participants initially consented to recontact, our results highlighted the importance of tailored consent procedures that clearly inform participants about the possibility of receiving actionable SFs and allow them to opt in or out of such disclosure [26]. This is especially crucial in research settings, where participants may not anticipate receiving clinical information. Furthermore, the integration of genomic-based programs into healthcare systems in Middle Eastern populations requires a careful consideration of various ethical and social issues [27].

In this pilot study, 10 high-risk participants were identified (6 females, 4 males); 2 female participants had their variants reclassified as VUS. The remaining eight participants had P/LP *BRCA1/2* variants confirmed and were referred to the cancer genetics and high-risk surveillance program. Most of the participants opted for increased surveillance rather than prophylactic surgeries due to several factors, including age, gender, and marital status. One female participant opted for prophylactic BSO and was found to have early-stage ovarian cancer (high-grade invasive serous carcinoma of the fallopian tube with serous tubal intraepithelial carcinoma, FIGO stage IA), which is a common ovarian cancer subtype associated with the *BRCA1* gene [28]; this was followed by adjuvant chemotherapy with excellent clinical outcomes, and the patient has remained disease-free post-surgery, with no recurrence reported at her last follow-up.

At the cancer genetics program, cascade testing of the index family members was offered, with a full acceptance rate in which a total of 20 positive family members were identified. All family members opted for increased surveillance, except for one family member (sister of index case who was found to have early-stage ovarian cancer post-prophylactic BSO) who chose to undergo a total abdominal hysterectomy with bilateral salpingo-oophorectomy and was found to have early-stage uterine cancer, specifically serous endometroid carcinoma. Interestingly, serous endometroid carcinoma of the uterus has also been reported in relation to pathogenic variants in the *BRCA1* gene [29]. These results underscore the critical importance of the early identification of high-risk individuals, enabling timely interventions that can lead to the early detection or even prevention of life-threatening cancers such as ovarian cancer. One of the key challenges addressed in the present workflow was the interpretation and classification of *BRCA1/2* variants. In concordance with the ACMG/AMP classification system [18], we downgraded the classification of seven P/LP variants that were identified and classified by Saad and colleagues to VUS or LB, resulting in the exclusion of 17 participants at the initial filtering step. Moreover, the clinical scientist team reclassified the *BRCA1* c.4096+1G>C variant to VUS, providing information on its limited pathogenicity. Leveraging standardized guidelines, such as the ACMG/AMP criteria, and incorporating population-specific variant databases helped reduce uncertainty in variant interpretation and enabled focusing on truly actionable findings.

Despite the success of this workflow and its promising outcomes, the challenges associated with implementing such a process must be acknowledged. These include ethical and financial considerations, as well as long-term follow-up and integration with national healthcare systems, which remain key obstacles for future incorporation. Additionally, participant preferences regarding recontact and data sharing must continue to be respected and dynamically managed. From a clinical perspective, implementing risk-reducing strategies for unaffected individuals carrying genetic mutations imposes a substantial burden on clinical services, including radiology, surgery, diagnostic molecular laboratories, and psychological support, not only for participants but also their families. The newly established CCPMG center can facilitate the multidisciplinary coordination of these services, ensuring comprehensive care through tailored interventions, counseling, and follow-ups within an integrated genomic and clinical framework. A limitation of our pilot study is that we did not assess the economic cost-effectiveness of the proposed framework of returning *BRCA1/2* actionable findings to biobank participants. All QPHI participants received care at no charge through the biobank and the HMC hospital; therefore, the costs associated with implementing the framework were not evaluated. Further studies should consider the financial implications of such a design, including the potential effect on downstream intervention, feasibility, and sustainability of returning all medically actionable genetic findings. Additionally, in this pilot phase, we intentionally limited our analysis to *BRCA1* and *BRCA2* to assess the feasibility of returning research-derived, rather than clinically generated, results to biobank participants. Although other genes such as *PALB2*, *PTEN*, and *TP53* are also associated with hereditary breast cancer, evaluating them was beyond the scope of this initial feasibility assessment. In routine clinical care, individuals with a strong family history typically undergo multigene panel testing that includes these moderate- and high-risk genes; in our study, the two participants (#6 and #7) had already received such clinical testing, and their negative results were part of their clinical evaluation rather than the research return process we aimed to evaluate. We acknowledge the importance of these additional genes and note that future phases of this project will expand the analysis to include the broader set of ACMG-recommended secondary findings genes.

## 5. Conclusions

The implementation of a structured workflow for the return of *BRCA1/2* medically actionable findings represents a robust step toward translating genomic data into clinical intervention in biobank settings. This model lays the groundwork for the broader application of returning medically actionable SFs, facilitating a paradigm shift in translating precision medicine to improve the health outcomes of patients and their families.

Our initiative bridges the gap between genomic research and clinical application, illustrating the promise of personalized medicine in improving health outcomes. Building on these results, we recommend extending this translational pathway to other actionable gene mutations.

## Figures and Tables

**Table 1:** BRCA1 and BRCA2 validated genetic findings.

Gene	HGVSc	HGVSp	Initial Variant Class	Confirmed Variant Class	GPP	HET	HOMO
BRCA 1	c.4787C>A	p.Ser1596Ter	P	P	2	2	0
BRCA 1	c.1365dup	p.Ile456fs	LP	LP	2	2	0
BRCA 1	c.4096+1G>C	NA	LP	VUS	2	2	0
BRCA 1	c.4065_4068del	p.Asn1355fs	P	P	1	1	0
BRCA 2	c.4211_4215del	p.Ser1404Ter	P	P	2	2	0
BRCA 2	c.-39-1G>C	NA	LP	LP	1	1	0

Abbreviations: P, pathogenic; LP, likely pathogenic; VUS, variants of uncertain significance; GPP, genotype-positive participants; HET, heterozygous; HOMO, homozygous.

**Table 2:** Clinical characteristics of the pilot cohort.

Pts-ID	Gene	HGVSc	HGVSp	Zygosity	Sex	Age at Testing	Parents Consanguinity	Related Personal Hx	Family Hx of Cancer	Final Variant Class	Surveillance Decision	Risk-Reducing Strategy
#1	BRCA1	c.4787C>A	p.Ser1596Ter	HET	M	64	No	No	Strongly positive	P	Referred to the high-risk surveillance	Under surveillance consistent with recommendations for male carriers
#2	BRCA2	c.4211_4215del	p.Ser1404Ter	HET	M	72	No	No	No	P	Referred to the high-risk surveillance	Under surveillance consistent with recommendations for male carriers
#3	BRCA2	c.4211_4215del	p.Ser1404Ter	HET	M	42	No	No	Positive	P	Referred to the high-risk surveillance	Under surveillance consistent with recommendations for male carriers
#4	BRCA1	c.1365dup	p.Ile456fs	HET	F	26	No	No	Strongly positive	LP	Referred to the high-risk surveillance	Under surveillance consistent with recommendations for young female carriers
#5	BRCA1	c.4065_4068del	p.Asn1355fs	HET	F	57	No	No	Positive	P	Referred to the high-risk surveillance	Under surveillance for breast cancer and opted in for risk-reducing salpingo-oophorectomy
#6	BRCA1	c.4096+1G>C	NA	HET	F	52	No	Recurrent fibroids, hysterectomy, and salpingectomy	Positive	VUS	Discharge with no further action	NA
#7	BRCA1	c.4096+1G>C	NA	HET	F	40	First cousin	No	Positive	VUS	Discharge with no further action	NA
#8	BRCA1	c.4787C>A	p.Ser1596Ter	HET	M	44	No	No	Strongly positive	P	Referred to the high-risk surveillance	Under surveillance consistent with recommendations for male carriers
#9	BRCA2	c.-39-1G>C	NA	HET	F	53	No	No	No	LP	Referred to the high-risk surveillance	Under surveillance for breast cancer
#10	BRCA1	c.1365dup	p.Ile456fs	HET	F	39	No	No	Strongly positive	LP	Referred to the high-risk surveillance	Under surveillance consistent with recommendations for young female carriers

**Abbreviations:** Pts-ID, participant identification number; P, pathogenic; LP, likely pathogenic; VUS, variants of uncertain significance; HET, heterozygote carriers; Hx, history.

## Data Availability

The data used in this study are available from the QPHI. However, access to these data is restricted, as they were used under license QF-QBB-RES-ACC-0241 for the purposes of the current research and are not publicly accessible. Data may be made available from the authors upon reasonable request and with the approval of the QBB Institutional Review Board.

## Supplementary Materials

The following supporting information can be downloaded at https://www.mdpi.com/article/10.3390/biomedicines13123047/s1.

## Abbreviations

The following abbreviations are used in this manuscript:

HBOC	Hereditary Breast and Ovarian Cancer Syndrome
P	Pathogenic
LP	Likely Pathogenic
VUS	Variants of Uncertain Significance
QPHI	Qatar Precision Health Institute
GPPs	Genotype-Positive Participants
HMC	Hamad Medical Corporation
GS	Genome Sequencing
SFs	Secondary Findings
ACMG	American College of Medical Genetics and Genomics
NCCCR	National Center for Cancer Care and Research
CCPMG	Centre of Clinical Precision Medicine and Genomics
EMRs	Electronic Medical Records
NCCN	National Comprehensive Cancer Network
