# Supplementary material for: From Biobank to Bedside: A Pilot Study on Returning Medically Actionable BRCA1/2 Results in Qatar’s Precision Medicine Landscape
Source: Biomedicines. 2025 Dec 11;13(12):3047. doi: 10.3390/biomedicines13123047 (PMC12731134; doi:10.3390/biomedicines13123047)
Supplement: Supplementary file 1 [file biomedicines-13-03047-s001.zip › biomedicines-4005941-supplementary.pdf]

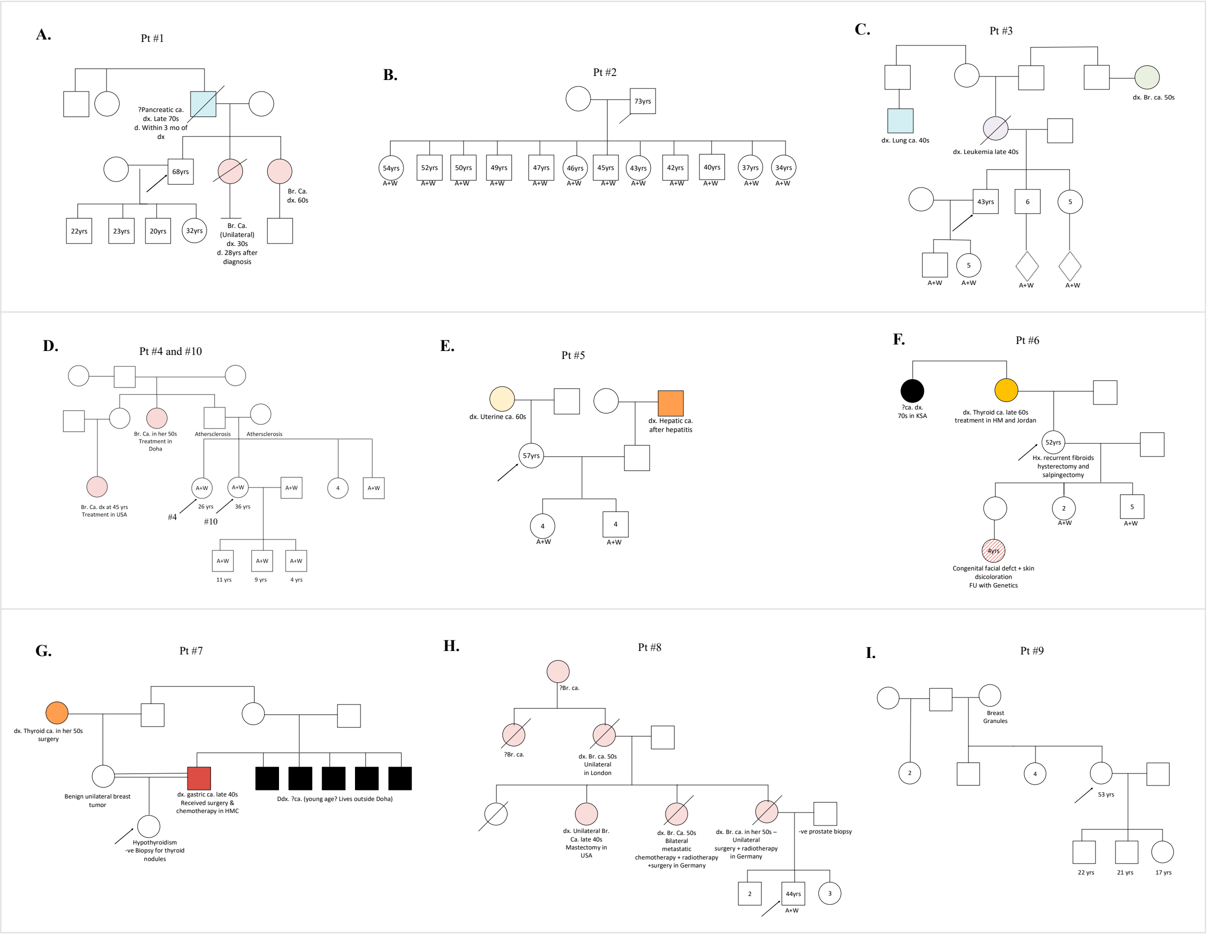

Supplementary Figure S1. Pedigrees and Family Histories of Pilot Study Participants. **A.** participant number 1, **B.** participant number 2, **C.** participant number 3, **D.** participant number 4 and number 10 (siblings), **E.** participant number 5, **F.** participant number 6, **G.** participant number 7, **H.** participant number 8, **I.** participant number 9. **Abbreviations:** Pt, participants; ca, cancer; dx, diagnosis.

Supplementary Table S1. BRCA1 and BRCA2 List of Pathogenic and Likely Pathogenic Variants Identified in the Qatar Genome Research Study.

Gene	HGVSc	HGVSp	Transcripts	HET	HO	VEP Most Severe Consequence	CharGer Classification	ClinVar CLNSIG	Variant reclassification per ACMG criteria
BRCA1	c.4787C>A	p.Ser1596Ter	NM_007294.4	4	0	Stop gained	Pathogenic	Pathogenic	Pathogenic
BRCA1	c.1140dup	p.Lys381fs	NM_007294.4	2	0	Frameshift variant	Pathogenic	Pathogenic	Pathogenic
BRCA1	c.5096G>A	p.Arg1699Gln	NM_007294.4	1	0	Missense variant	Likely pathogenic	Pathogenic	Pathogenic
BRCA1	c.4065_4068del	p.Asn1355fs	NM_007294.4	1	0	Frameshift variant	Pathogenic	Pathogenic	Pathogenic
BRCA1	c.4186-2A>G	NA	NM_007294.4	1	0	Splice acceptor variant	Pathogenic	Pathogenic	VUS
BRCA1	c.4136_4137del	p.Ser1379Ter	NM_007294.4	1	0	Frameshift variant	Pathogenic	Pathogenic	Pathogenic
BRCA1	c.2376G>A	p.Gly792=	NM_007294.4	4	0	Synonymous variant	Pathogenic	Likely benign	Likely benign
BRCA1	c.1365dup	p.Ile456fs	NM_007294.4	4	0	Frameshift variant	Pathogenic	NA	Likely pathogenic
BRCA1	c.4096+1G>C	NA	NM_007294.4	3	0	Splice donor_variant	Pathogenic	NA	Likely pathogenic
BRCA1	c.4358-2787A>T	NA	NM_007294.4	3	0	Splice acceptor variant	Pathogenic	NA	VUS
BRCA2	c.4211_4215del	p.Ser1404Ter	NM_000059.4	4	0	Frameshift variant	Likely pathogenic	Pathogenic	Pathogenic
BRCA2	c.3751dupA	p.Thr1251fs	NM_000059.4	2	0	Frameshift variant	Likely pathogenic	Pathogenic	Pathogenic
BRCA2	c.3847_3848del	p.Val1283fs	NM_000059.4	1	0	Frameshift variant	Likely pathogenic	Pathogenic	Pathogenic
BRCA2	c.6405_6409del	p.Asn2135fs	NM_000059.4	1	0	Frameshift variant	Pathogenic	Pathogenic	Pathogenic
BRCA2	c.9382C>T	p.Arg3128Ter	NM_000059.4	1	0	Stop gained	Pathogenic	Pathogenic	Pathogenic
BRCA2	c.5557dup	p.Cys1853fs	NM_000059.4	1	0	Frameshift variant	Likely pathogenic	Pathogenic	Pathogenic
BRCA2	c.2760C>A	p.Pro920=	NM_000059.4	3	0	Synonymous variant	Pathogenic	NA	Likely benign
BRCA2	c.-39-1G>C	NA	NM_000059.4	2	0	Splice acceptor variant	Likely pathogenic	NA	Likely pathogenic
BRCA2	c.3837T>C	p.Asn1279=	NM_000059.4	1	0	Synonymous variant	Pathogenic	Likely benign	Likely benign
BRCA2	c.151G>A	p.Glu51Lys	NM_000059.4	3	0	Missense variant	Likely pathogenic	VUS	VUS
BRCA2	c.9924C>T	p.Tyr3308=	NM_000059.4	2	0	Synonymous variant	Likely pathogenic	Benign	Benign
BRCA2	c.2711_2714dup	p.Asn905fs	NM_000059.4	2	0	Frameshift variant	Likely pathogenic	NA	Pathogenic

Abbreviations: P, pathogenic; LP, likely pathogenic; VUS, variants of uncertain significance; HET, heterozygous; HOMO, homozygous; NA, not available.
